# Supplementary material for: Narrow genetic diversity in germplasm from the Guinean and Sudano-Guinean zones in Benin indicates the need to broaden the genetic base of sweet fig banana (Musa acuminata cv Sotoumon)
Source: PLoS One. 2023 Nov 16;18(11):e0294315. doi: 10.1371/journal.pone.0294315 (PMC10653437; doi:10.1371/journal.pone.0294315)
Supplement: S1 Table — This is a word file presenting the coordinates ranges of the studied banana populations. (DOCX) [file pone.0294315.s001.docx]

**S1** **Table** Geographical situation of the eight Sweet fig banana populations.

| Population code | Geographic locations | | | | | Sampling size |
| --- | --- | --- | --- | --- | --- | --- |
|  | Depart-ment | Districts | Latitude | Longitude | Altitude (m) |  |
| **Guineo-Congolian agro-ecological zone** | | | | | | |
| ZTA | Atlantic | Zè | N 6° 47′ 00″ | E 2°1 8′ 00″ | 35 | 14 |
|  |  | Tori-Bossito | N 6° 30′ 11″ | E 2° 08′ 42″ | 42 | 12 |
|  |  | Allada | N 6°39′55″ | E 2°09′ 04″ | 46 | 27 |
| SAD | Plateau | Sakété | N 6° 44′ 11″ | E 2° 39′ 29″ | 62 | 10 |
|  | Ouémé | Adjohoun | N 6° 42′ 43″ | E 2° 29′ 38″ | 79 | 10 |
| LOA | Mono | Lokossa | N 6° 38′ 00″ | E 1° 43′ 00″ | 31 | 09 |
|  |  | Athiémè | N 6° 34′ 60″ | E 1° 40′ 00″ | 9 | 25 |
| GPO |  | Grand-Popo | N 6° 15′ 50″ | E 1° 44’4″ | 4 | 27 |
| DOL | Couffo | Dogbo | N 6° 49′ 00″ | E 1° 47′ 00″ | 68 | 18 |
|  |  | Lalo | N 6° 55′ 00″ | E 1° 53′ 00″ | 80 | 09 |
| ZOO | Zou | Zogbodomey | N 7° 4′ 60″ | E 2° 6′ 00″ | 70 | 16 |
|  |  | Ouinhi | N 7° 05′ 00″ | E 2° 29′ 00″ | 36 | 27 |
| **Sudano-Guinean agro-ecological zone** | | | | | | |
| BBG | Collines | Bantè | N 8° 25′ 0″ | E 1° 53′ 33″ | 291 | 18 |
|  |  | Glazoué | N 7° 58′ 25″ | E 2° 14′ 24″ | 183 | 22 |
|  | Donga | Bassila | N 9° 0′ 9″ | E 1° 40′ 70″ | 283 | 18 |
| PST | Collines | Savè | N 8° 1′ 48″ | E 2° 29′ 24″ | 177 | 5 |
|  | Borgou | Parakou | N 9° 21′ 0″ | E 2° 37′ 00″ | 369 | 2 |
|  |  | Tchaourou | N 8° 52′ 60″ | E 2° 36′ 00″ | 323 | 4 |

Population code: GPO: Grand-Popo ; LOA: Lokossa, Athiémè ; DOL: Dogbo, Lalo ; ZTA: Zè, Tori-Bossito, Allada ; SAD: Adjohoun, Sakété ; ZOO: Zogbodomey, Ouinhi ; BBG: Bantè; Bassila, Glazoué; PST: Parakou; Savè, Tchaourou
